# MEETINGS CALENDAR - 2018

**Published:** 2017

## January

### ► 11 and 12 - 1^st^ International Conference - Sublobar Resections for Lung Cancer

Paris, France

Informations: Madinina Cox

Phone: +33 (0)6 35 31 40 90

E-mail: madinina.cox@mc-com.fr

Site: conference-thoraxparis.com/

### ► 14 to 19 - The 36^th^ Annual International Symposium: Anesthesiology, Surgery and Perioperative Medicine

Paradise Island, Bahamas

Informations: George Silvay

E-mail: george.silvay@mountsinai.org

Site: www.clinicalupdateinanesthesiology.org

### ► 25 - Toronto Cardiac Tumours Conference

Toronto, Canada

Informations: Continuing Professional Development

Site:www.cpd.utoronto.ca/cardiactumours/

### ► 26 and 27 - Better Breathing Conference 2018

Toronto, Canada

Informations: Amy Rice

Phone: 416864-9911

E-mail: arice@lungontario.ca

Site: www.betterbreathing.ca/

### ► 27 to 31 - STS 54^th^ Annual Meeting

Fort Lauderdale, United States

Informations: STS

Phone: 312-202-5800

Fax: 312-202-5801

E-mail: education@sts.org

Site: sts.org/annualmeeting

## February

### 31 to 2 - Lung Transplantation Hands - On Course HUPHM

Madrid, Spain

Information:

Site: www.cursotrasplantepulmonar.com/

### ► 5 to 9 - Fundamentals in Cardiac Surgery: Part I

Windsor, United Kingdom

Informations: EACTS

E-mail: info@eacts.co.uk

Site: www.eacts.org

### ► 15 to 17 - The Houston Aortic Symposium: Frontiers in Cardiovascular Diseases, the Eleventh in the Series

Houston, United States

Informations: Rebecca Law

Phone: 7607202263

Fax: 7607206263

E-mail: rlaw@promedicacme.com

Site: www.houstonaorticsymposium.com/

### ► 19 and 20 - 15^th^ International Conference On Pediatrics and Pediatric Cardiology

Paris, France

Informations: Sharon Williams

Phone: 7025085200

Fax: 7025085200

E-mail: pediatriccardiologists@pediatricsconferences.org

Site: pediatriccardiology.conferenceseries.com/europe/

### ► 19 to 21 - 23^rd^ European Heart Disease and Heart Failure Congress

Paris, France

Informations: Cathy Smith

E-mail: heartfailure@cardiologyconference.org

Site: heartdiseases.conferenceseries.com/

### ► 22 and 23 - Endoscopic Port-Access Mitral Valve Repair Drylab Training

Maastricht, Netherlands

Informations: EACTS

E-mail: info@eacts.co.uk

Site: www.eacts.org

